# Longer trinucleotide repeats of androgen receptor are associated with higher testosterone and low oxytocin levels in diabetic premature ejaculatory dysfunction patients

**DOI:** 10.1186/s12610-018-0068-0

**Published:** 2018-03-06

**Authors:** Haroon Latif Khan, Shahzad Bhatti, Sana Abbas, Yousaf Latif Khan, Rosa Maria Marquez Gonzalez, Muhammad Aslamkhan, Gerardo Rodriguez Gonzalez, Hikmet Hakan Aydin

**Affiliations:** 1 Lahore Institute of fertility and Endocrinology, Hameed Latif Hospital, 14 – Abu Bakar Block, New Garden Town, Lahore, Pakistan; 2 Department of Human Genetics and Molecular biology, University of Health Sciences, Lahore, -54600 Pakistan; 3 Institute of Molecular Biology and Biotechnology, The University of Lahore, Lahore, 54600 Pakistan; 4 Department of Medical Education, Rashid Latif Medical College, Lahore, Pakistan; 5 Centro de investigacion Biomedica de Occidente, IMSS, Uiversidad de Guadalajara, Jalisco Maxico, Guadalajara, Mexico; 6 Universidad De Guadalajara CIBO, IMSS, Sierra Mojada 800 Independencia, 44340 Guadalajara, Jalisco Maxicom Mexico; 7 Department of Medical Biochemistry, Ege University School of Medicine, Bornova, Izmir Turkey

**Keywords:** Androgen receptor, Testosterone, Oxytocin, Premature ejaculation, Diabetes mellitus, Récepteur aux androgènes, Testostérone, Ocytocine, Ejaculation prématurée, Diabète de type 2

## Abstract

**Background:**

Despite its worldwide high occurrence, the obscurity regarding the description, epidemiology and management of premature ejaculation remains provocative. It is well established that male premature ejaculatory dysfunction is an increasing problem due to spontaneous ejaculation across a variety of general and clinical subjects. The main goal of this study was to determine the relationships between trinucleotide repeats of the androgen receptor (AR), sex steroids, and pituitary hormones with sexual function in men with type 2 diabetes mellitus (DM) and reported with acquired premature ejaculation (PE).

**Methods:**

A total of 150 normal and 250 PE + DM subjects were enrolled in this study. Each subject was invited to fill out an elaborative questionnaire to acquire precise selective information regarding BMI, duration of PE + DM, self-reported Intra-Vaginal Ejaculatory Latency Time (IELT), sexual and mental health status by using the premature ejaculation diagnostic tool (PEDT) and Beck Depression Inventory-II (BDI-II). Pearson’s correlation analysis was used to analyze the relationship between clinical, hormonal, and genetic variables. Ward’s minimum variance cluster analysis and principal component analysis were used for evaluation of dependence between genetic, clinical, and demographic parameters.

**Results:**

The patients who have the lowest number of (≤21) (CAG)n repeats have higher serum oxytocin levels (114.2 pg/ml; *n* = 54, 43.2%) than the controls (69.18 pg/ml; *n* = 22, 17.6%) and the patients with the highest (≥26) number of (CAG)n repeats (62.9 pg/ml; *n* = 108, 43.2%).

On the other hand, patients who have the highest numbers of (CAG)n repeats (≥26) have higher serum testosterone (6.1 ng/ml; *n* = 108, 43.2% of cohort) lower prolactin (3.01 ng/ml; *n* = 108, 43.2% of cohort) levels than the controls and patients with the lowest numbers (≤21) of (CAG)n repeats and their TSH (1.53 mIU/L, *P* < 0.05) levels are lower than those of controls. In the Pearson correlation model, self-estimated IELT demonstrated significantly negative correlation with both (CAG)n and (GCC)n repeats (*r* = − 0.16, *p* = 0.0001; *r* = − 0.19, *p* = 0.0001) respectively. These repeats have positive correlation with PEDT (*r* = 0.28, *p* = 0.0001: *r* = 0.24, *p* = 0.0001, whole model) and inversely correlated with BDI-II (*r* = − 0.25, *p* = 0.0001).

**Conclusion:**

This study indicates that androgen receptor polymorphism modulates the endocrine effect on ejaculatory reflex and depends strongly on its “cofactors”. Moreover, our results also confirmed an association between long tri-nucleotide repeats of androgen receptor, sex steroids, pituitary, and thyroid hormones in relation to  acquired premature ejaculatory dysfunction in diabetic patients. However, endocrine regulation of PE reflex is a complex phenomenon that requires further investigation.

## Résumé

**Contexte:**

Malgré une fréquence élevée partout dans le monde de l’éjaculation prématurée (EP), le caractère obscur de sa description, de son épidémiologie et de sa prise en charge reste provocateur. Il est avéré que la dysfonction masculine représentée par l’EP est un problème croissant en raison de l’occurrence de l’éjaculation spontanée dans de nombreux sujets généraux et cliniques. L’objectif principal de la présente étude était de déterminer les relations entre le nombre de répétions de trinucléotides du récepteur aux androgènes (RA), les stéroïdes sexuels et les hormones hypophysaires d’une part, et la fonction sexuelle d’hommes qui présentent un Diabète de type 2 (DT2) et qui rapportent une EP acquise.

**Sujets et Méthodes:**

Un total de 150 sujets normaux et de 250 sujets qui présentaient une EP et un DT2 ont été enrôlés dans cette étude. Il a été demandé à chaque sujet de remplir un questionnaire approprié au recueil sélectif d’informations précises concernant l’indice de masse corporelle, la durée de l’EP+ DT2, le temps de latence éjaculatoire intra vaginal (IELT) auto-rapporté, ainsi que les statuts sexuel et mental sur la base de l’outil diagnostic de l’éjaculation prématurée (PEDT) et de l’inventaire de dépression de Beck-II (BDI-II). Les coefficients de corrélation de Pearson ont été utilisés pour évaluer les relations entre les paramètres génétiques, cliniques et démographiques. L’analyse de variance minimale des groupements de Ward et l’analyse en composante principale ont été utilisées pour évaluer la dépendance entre les paramètres génétiques, clinique et démographiques.

**Résultats:**

Les sujets qui avaient le plus faible nombre (≤21) de répétitions de (CAG)n présentaient des taux sériques plus élevés d’ocytocine (114.2 pg/ml; n = 54, 43.2%) que les témoins (69.18 pg/ml; n = 22, 17.6%) et que les sujets avec le nombre le plus élevé (≥26) de répétitions de (CAG)n (62.9 pg/ml; n = 108, 43.2%).

D’un autre côté, les sujets qui présentaient le nombre le plus élevé (≥26) de répétitions de (CAG)n avaient des taux sériques de testostérone plus élevés (6.1ng/ml; n = 108, 43.2% de la cohorte) et de prolactine plus bas (3.01ng/ml; n = 108, 43.2% de la cohorte) que les témoins et que les sujets qui présentaient le nombre le plus bas (≥21) de répétitions de (CAG)n; et leurs taux sériques de TSH était plus bas (1.53 mIU/L; p < 0.05) que ceux des témoins. Dans le modèle de corrélation de Pearson, l’IELT auto-rapporté présentait une corrélation négative avec les répétitions à la fois des triplets (CAG)n (r = -0.16, p = 0.0001) et des triplets (GGC)n (r = -0.19, p = 0.0001). Ces répétitions étaient respectivement corrélées positivement avec PEDT (r = 0.28, p = 0.0001; r = 0.24, p = 0.0001, modèle global) et inversement corrélées avec BDI-II (r = -0.25, p = 0.0001).

**Conclusion:**

Cette étude montre que le polymorphisme du récepteur aux androgènes module l’effet endocrinien sur le reflexe éjaculatoire et qu’il est étroitement dépendant de ses « cofacteurs ». De plus, les présents résultats confirment aussi l’association entre les longues répétions de trinucléotides du récepteur aux androgènes, les stéroïdes sexuels, les hormones pituitaires et thyroïdiennes en relation avec une dysfonction éjaculatoire prématurée acquise chez les patients diabétiques. La régulation endocrine du réflexe de l’EP est toutefois un phénomène complexe qui nécessite de futures investigations.

## Background

The control of male ejaculatory function is a mechanistic, self-efficacious, multifunctional process comprising inputs of the sympathetic neuronal system, interconnected contractions of ductus deferens and reduced resistance to ductus ejaculatory closure [1]. An ejaculatory dysfunction is a heterogeneous group of dysfunctions that might be an outcome of interruption at any point in the sequential cascade of discharge, evacuation and sexual climax [2].

Among men, premature ejaculation (PE) or premature climax is the most prevalent form that has the greatest impact on the male sexual dysfunction and referred as a culture based symptom of the society that is self-described, self-distinguished and self-ranked [3, 4]. It is a condition of short ejaculatory latency, characterized by rapid recurrent ejaculation with insignificant sexual motivation prior, on or soon after heterosexual vaginal penetration or lack of control upon ejaculation [5]. PE classification system has been broadly established as primary PE (Lifelong PE) and secondary PE (acquired during the life). Primary PE presents since a start from the sexual activity with nearly every sexual partner and also encountered during self-masturbation [6]. While, acquired PE is the most common condition, with high prevalence rate (30%) and frequently reported in different epidemiological studies [7, 8].

The updated guidelines for PE proposed by International Society for Sexual Medicine (ISSM) are based on three critical points I) Brief Intra-Vaginal Ejaculatory Latency Time (IELT), spans from penetration to ejaculation i.e. a short involuntary ejaculatory episode that should be less than one minute; however, IELT that spans from 1 to 2 min is considered short if it exerts significant distress on both partners or one of them. II) Loss of self-control about the timing of ejaculation or inability to control the onset of ejaculation III) Un-satisfaction, fear, and severity of psychological distress related to ejaculatory dysfunction [9].

Androgen receptor (AR) is likely responsible for androgen mediating signaling and plays a key role in the sexual differentiation during embryonic development, and growth of secondary sex characters [10]. Human AR gene is located on the long arm of X-chromosome (q11–12). It consists of 8 exons which are more than 90 kb long and transcribed into fully functional protein of about 919 amino acids [11]. Androgen receptor gene has two polymorphic tandem repeats characterized by varied numbers of (*CAG*)n and (*GGC*)n repeats, located on the opposite ends of exon 1. AR protein functions as an androgen hormone- activated transcription factor which consists of three functional domains: N-terminal transactivation domain (NTD), DNA binding domain (DBD) and Ligand binding domain (LBD). The NTD of the AR protein harbor varied numbers of poly-glutamine (poly-Q) and poly-glycine (poly-G) stretches, which are transcribed by the (*CAG*)n and (*GGC*)n repeats [12]. On binding the ligand, AR separates from accessory proteins, translocates into the nucleoplasm, dimerizes and starts the transcription of the AR- responsive elements [13]. Longer (*CAG*)n repeats are responsible for compromised AR transcriptional activity whereas, very short repeats are the leading cause of prostate cancer, however, the role of the long (*GGC*)n repeats is still uncertain [14].

The obligatory role of endocrine regulation in acquired PE has been well-known over the past decades. The androgens and hypophyseal hormones have been considered as potentially important candidates in the modulation of ejaculatory mechanism [15]. Among androgens testosterone (TT) plays a pivotal role in the maintenance of accessory organs and is involved specifically in the regulation of normal spermatogenesis. It affects  the control of ejaculatory mechanism both on central and peripheral levels. It also has a facilitatory role in the control of ejaculatory reflex, sexual behavior and copulation frequency [16].

Diabetes mellitus (DM) and thyroid disorders are believed to play a vital role in the development of PE, both describe the two most recurrent endocrinopathies found in clinical practice. Moreover, these disorders trigger the lifelong adverse effects on self-perception and mental health of men by altering the central serotoninergic pathways, resulting in the diminished ejaculation control practice [17]. A recent study has pointed out that PE may be differentially affected by pituitary hormones such as oxytocin, prolactin as well as thyreotropin at various levels of ejaculatory process [18]. Therefore, the aim of present study is to investigate any possible association between tri-nucleotide repeats of androgen receptor, sex steroids and pituitary hormones in a cohort of diabetic patients consulting for premature ejaculatory dysfunction.

## Methods

### Subjects

A total of 250 diabetic patients consulting for premature ejaculatory dysfunction were selected: 120 outpatients diagnosed with mild to moderate comorbid erectile dysfunction (ED). Firstly, these patients were treated for ED but when they continue to have PE, they were included in this study. Control group consists of 150 non-diabetic subjects with normal IELT (> 4 min) and normal International index of erectile function (IIEF-15) score.

The study was approved by the Institutional Ethical Committee (IEC) in accordance with Helsinki Declarations. Personal interviews were conducted to get the information regarding patient’s age, onset and duration of PE, self-reported Intra-Vaginal Ejaculatory Latency Time (IELT) and prevalence of any congenital disease. Sexual and mental health status was evaluated by using the premature ejaculation diagnostic tool (PEDT) and Beck Depression Inventory-II (BDI-II), after obtaining consent from each contributor. Moreover, a clinical investigator physically examined the subjects before taking blood samples.

#### Inclusion criteria

Subjects between the age of 30 to 60 years were selected, according to the following criteria: i) normal urine albumin to creatinine ratio (< 30 mg/g) on spot urine, ii) normal ejaculation experiences iii) acquired PE for at least more than six months iv) monogamous v) detailed drug treatment and had no cause of organic PE vi) devoid of anatomical anomalies, vii) experience morning stiffness with no history of using selective serotonin reuptake inhibitors viii) continuous sexual relationship for at least twelve months ix) trying for intercourse more than once per week x) had no psychiatrical disorder and absent perceived ejaculatory control, met the DSM-IV-TR criteria for PE, had IELT ≤3 min [19].

#### Exclusion criteria

Patients with any congenital defect, hypogonadism, TSH levels outside reference range (0.4–4.5 mIU/L), urogenital surgery, chronic prostatitis, any sexually transmitted disease, habitual to use ejaculation delaying pills, sprays, condoms, psychological and mental illness, alcoholic addiction, and infectious disease were excluded from the study. Moreover, those patients whose female partners had anorgasmia, inactive sexual desire, and pain (vaginismus) disorders were not included in this study.

### Assessment of the polymorphic (*CAG*)n and (*GGC*)n repeats copy number

Genomic DNA was extracted from peripheral blood by using revised salting out method [20]. AR exon 1 repeats (*CAG*)n and (*GGC*)n were amplified from genomic DNA in 50 μl reaction mixture, having 10 ng genomic DNA,10 μM (2.5 mmol) Mgcl2, 0.5 μl of each primer and AmpliTaq Gold® (Applied Bios systems, USA) master mix were used according to the manufacturer’s instructions. Amplification of (*CAG*)n repeats has been done by forward primer sequence GTGGTTGCTCCCGCAAGTTTCC and reverse primer GCTCCCACTTCCTCCAAGGACAATTA. While the automated sequencing has been done through GCTGTGAAGGTTGCTGTTCCTC; for (*GGC*)n repeats forward primer was CAGCAAGAGACTAGCCCCAG and reverse was CCAGAACACAGAGTGACTCTGCC. (*GGC*) repeats sequenced with primer GGACTGGGATAGGGCACTCTGCTCAACC [21]. PCR extension was achieved using BioRad™ MJ Mini thermal cycler (Applied Bios systems, USA) under standard conditions: initial denaturation at 95 °C for 10 min, followed by 35 cycles of 94 °C for 3 min, annealing at 59 °C for 30 s, extension at 72 °C for 1 min and final extension at 72 °C for 10 min. ExoSAP-IT® (USB, Cleveland, OH, USA) was employed for refinement of PCR product. Sequencing was carried out by 3500 Genetic Analyzer (Applied Biosystems, Inc.) by using Big DyeTM Terminator version 3.1 cycle sequencing kit (Applied Bio systems) according to the manufacturer’s instructions.

### Estimation of clinical parameters

Clinical parameters such as height and weight were recorded to compute the body mass index (BMI) according to standard protocol [22]. Glycated hemoglobin (HbA_1c_) was determined by Menarini Analyzer (Hb 9210 Menarini Diagnostics, UK).

### Valuation of endocrine dimensions

Blood samples were collected between 8 to 10 am from cubital vein and serum was separated instantly and stored at − 20 °C till the performance of hormonal assays including oxytocin, prolactin, testosterone, and TSH through electrochemiluminenscence immunoassay, in accordance with manufacturer’s instructions (Elecsys® Roche Diagnostics, Indianapolis, USA).

### Evaluation of sexual and mental performance

Sexual performance was estimated according to the International Index of Erectile Function-15 (IIEF-15), which includes 5 domains: erectile function (items; 1, 2,3,4,5 and 15), sexual desire (items; 11 and 12), intercourse satisfaction (items; 6–8), overall satisfaction (items; 13and 14). According to the score, erectile function (EF) can be normal = 26–30, slightly impaired = 17–25, moderately impaired = 11–16, or severely impaired ≤11. Other parameters were evaluated, such as sexual desire (SD) ranging from score 2–10, intercourse satisfaction (IS) estimated from 0 to 15, Orgasm (OR) ranging 0–10 and overall satisfaction (OS) ranging from 2 to 10 score [23]. The incidence of PE is being determined according to the guidelines provided by The European Association of Urology (EAU). PEDT was assessed by using a questionnaire including five items, minimal stimulation, distress, interpersonal difficulties, self-control, and frequency. A score of ≤9 excludes PE [24]. The Degree of depression was calculated by BDI-II (Beck Depression Inventory-II) questionnaire according to following scoring criteria, 0–13 = minimal range, i.e. no depression, 14–19 = mild depression, 20–28 = moderate depression, 29–63 = severe depression [25].

### Statistical analysis

Data are presented as mean with standard deviation and as median with interquartile range (IQR) for subgroups due to the number of patients. Clinical parameters, hormonal assays, premature ejaculation assessments, depression assessments and sexual assessments were compared between the PE + DM group and the control group using the nonparametric Mann-Whitney test. Kruskal-Wallis / two-tailed test was performed to find the outcome differences in the distribution of values across the three subgroups: the short CAG (≤21), medium CAG (22–25) and long CAG (≥26) repeats of PE + DM patients and entire control samples. Pearson correlation analysis was used to analyze the relationships among clinical, hormonal, and genetic variables. Finally, the dependence between genetic, clinical, and demographic parameters was evaluated using Ward’s minimum variance cluster analysis and principal component analysis.

Statistical analysis was done using statistical package SPSS (version 21; SPSS Inch., Chicago, IL, USA) and XLSTAT 2017 software. The significant statistical difference was considered *p* < 0.05.

## Results

### Characteristics of the diabetic premature ejaculatory dysfunction patients

**Fig. 1:**
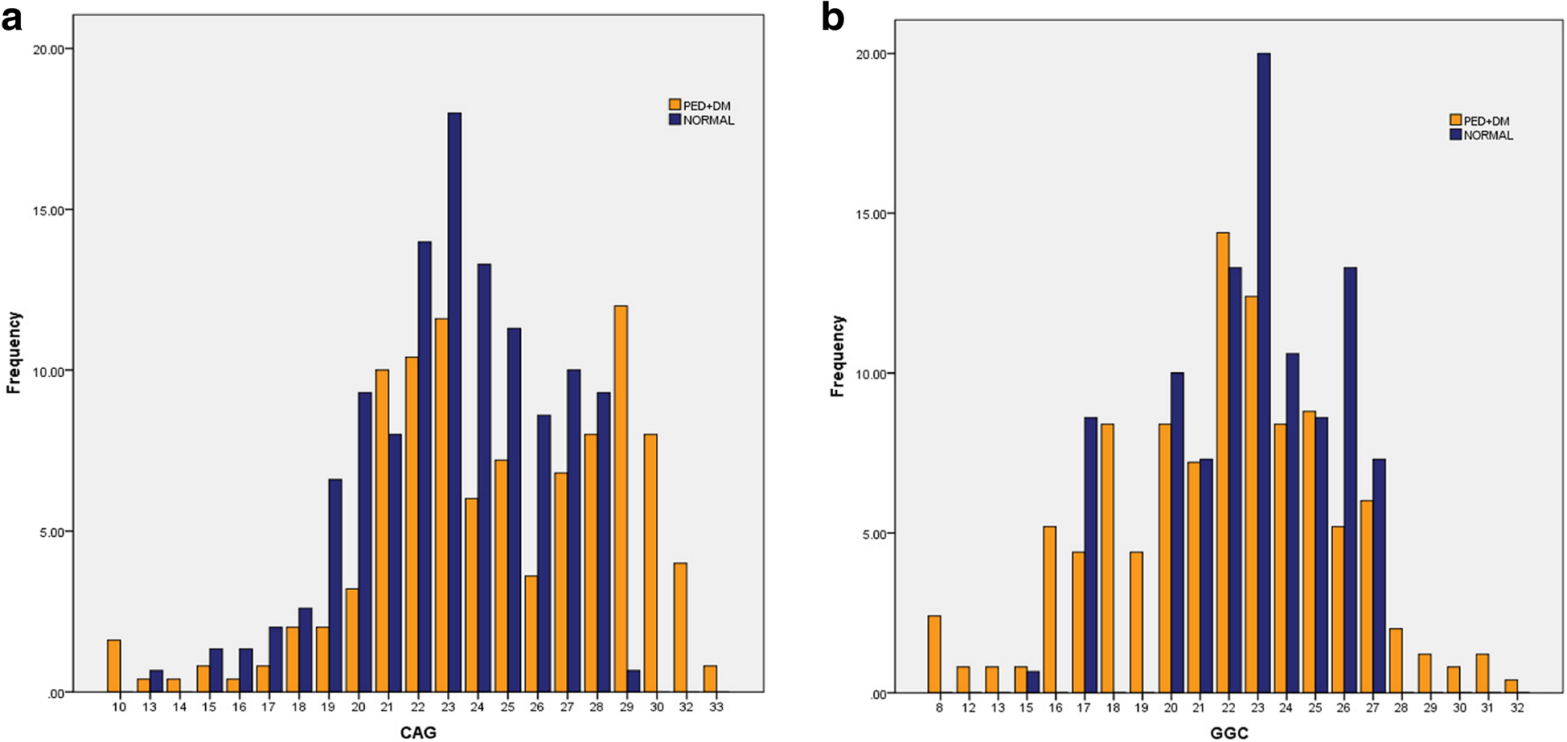
Sharing and prevalence of the Androgen receptor (AR) gene polymorphism. **a** (*CAG*)n repeats (**b**) (*GGC*)n repeats in diabetic premature ejaculatory dysfunction patients (*n* = 250) and normal subjects (*n* = 150)

The detailed characteristics of PE patients are given in the Table 1. Comparisons between patients and controls show significant differences. In addition to the expected difference regarding indicators of PE, patients have significantly higher oxytocin, lower PRL, and TSH respectively. Figure 1 shows the ranges of the number of (*CAG*)n and (*GGC*)n repeats in patients and controls, the highest and the lowest number of repeats are found among the patients. (*CAG*)n repeats range from 10 to 33 with a mean of 22.0 ± 5.34 and median of 22, whereas (*GGC*)n repeats range from 8 to 32 repeats with a mean of 22.01 ± 4.36 and median 22. The subjects with long (*CAG*)n repeats trend to have longer (*GGC*)n repeats. Mean age of PE subjects and control group was not different and ranged between 45.0 ± 10.1 to 45.3 ± 7.5 respectively. However, PE subjects were more likely to be obese (BMI = 31.5 ± 4.0) than control subjects (29.43 ± 3.99).

**Table 1:** Baseline demographics, Clinical and hormonal characteristics of diabetic premature ejaculatory dysfunction patients and normal study cohort

Variables	Subjects with PE + DM (*n* = 250) Mean ± SD	Control Subjects (*n* = 150) Mean ± SD	ρ*
Clinical Parameters			
(*CAG*)n repeats	22.0 ± 5.34	21.4 ± 6.3	NS
(*GGC*)n repeats	22.01 ± 4.36	22.0 ± 7.2	NS
Age (years)	45.0 ± 10.1	45.3 ± 7.5	NS
BMI (kg/m^2^)	31.5 ± 4.0	29.43 ± 3.99	NS
Blood collection time (8–10 AM)/30 mint	8.79 ± 2.59	9.29 ± 6.34	NS
HbA_1C_ (%)	7.01 ± 2.41	3.85 ± 1.05	< 0.001
Diabetes duration (years)	8.25 ± 4.59	–	–
Hormonal Assays			
Oxytocin (pg/ml)	87.9 ± 25.2	69.4 ± 23.6	< 0.001
Prolactin (ng/ml)	5.05 ± 2.31	7.1 ± 1.62	< 0.001
TSH (mIU/L)	1.56 ± 2.1	3.19 ± 1.6	< 0.001
Total Testosterone (ng/ml)	4.76 ± 1.52	3.63 ± 1.26	< 0.001
Premature Ejaculation Assessments			
PEDT	18.7 ± 3.81	7.21 ± 3.56	< 0.001
Self-estimated IELT (s)	121 ± 7.69	314.8 ± 28.36	< 0.001
Depression Assessments			
BDI-II	48 ± 9.40	8.7 ± 3.12	< 0.001
Sexual Assessments			
IIEF-15	44.3 ± 9.60	49.3 ± 2.8	NS

*PE*: Premature Ejaculatory dysfunction; *DM*: Diabetes mellitus type II; *NS* Not significant; *BMI* Body mass index; *TSH* Thyroid stimulating hormone; *PEDT* Premature ejaculation diagnostic tool; *BDI-II* Beck’s Depression Inventory-II; *IELT* Intravaginal ejaculatory latency time; *IIEF-15* International index of Erectile Dysfunction-15

Data have shown as mean ± SD. p* Mann-Whitney; *p* < 0.05 was considered statistically significant

### Longer trinucleotide (CAG)n repeats of the androgen receptor is associated with higher testosterone and low oxytocin

The categorization of the study subjects was shown in Table 2 that was based on the length of (*CAG*)n repeats i.e. short *CAG* (≤21), medium *CAG* ((22–25) and long *CAG* (≥26) repeats. The patients who have the lowest number of (≤21) (*CAG*)n repeats have higher serum oxytocin levels (114.2 pg/ml; *n* = 54, *p* < 0.05) than the controls (69.18 pg/ml; *p* < 0.05) and the patients with the highest (≥26) number of (*CAG*)n repeats (62.9 pg/ml; *n* = 108, Mann-Whitney U-test, *p* < 0.0001). On the other hand, patients who have the highest numbers of (*CAG*)n stretches (≥26) have higher serum testosterone (6.1 ng/ml; *n* = 108, *p* < 0.05) lower prolactin (3.01 ng/ml; *n* = 108, *p* < 0.05) and thyreotropin (1.53 mIU/L, *p* < 0.05) levels than the controls. There is also a significant difference with the other subgroups of patients, especially regarding IIEF-15 score in patients with long *CAG* (≥26) repeats, particularly in comparison with patients with short *CAG* (≤21).

**Table 2:** Clinical and sexual parameters of subjects with PE + DM and normal group based on Androgen receptor (*CAG*)n repeats

Subjects with PE + DM *n* = 250				Control subjects *n* = 150			
	CAG Long stretches ( ≥ 26) *n* = 108	CAG Medium stretches (22–25) *n* = 88	CAG short stretches ( ≤ 21) *n* = 54	Kruskal-Wallis = P*	CAG Long stretches ( ≥ 26) *n* = 43	CAG Medium stretches (22–25) *n* = 85	CAG short stretches ( ≤ 21) *n* = 22
Clinical Parameter							
Age (years)	45 (35–55)	43.5 (32–58)	45 (30–60)	NS	45 (30–60)	46 (32–60)	45 (30–60)
BMI (Kg/m2)	31 (30–32)	31.6 (29.3–33.9)	32.05 (30.1–34.0)	NS	31.1 (28.6–33.6)	31.3 (28.9–33.7)	31.9 (28.85–35.01)
Blood Sampling Time (8–10 Am)	9.00 (8.00–10)	8.65 (8.00–9.30)	8.72 (8.15–9.30)	NS	9.50 (9.00–10)	9.57 (9.15–10)	8.80 (8.30–9.30)
Diabetes duration (years)	8.30 (4.5–12.1)	7.85(2.2–13.5)	9.67 (6.37–12.98)	NS	9.3 (4.21–14.56)	8.4 (4.4–12.5)	7.5 (3.3–11.8)
HbA_1C_ (%)	6.95 (6–7.9)	6.95 (5.9–8.00)	7.1 (6.1–8.1)	< 0.0001	3.8 (3.2–4.5)	3.7 (3.1–4.3)	3.9 (3.3–4.6)
Hormonal Assays							
Oxytocin (pg/ml)	**62.9(43.1–82.8)**	**89.9 (69.9–99.8)**	**114.2 (99–129.5)**	< 0.0001	70.12 (58.1–82.1)	68.85 (56.4–81.3)	69.18 (57.1–81.2)
Prolactin (ng/ml)	**3.01 (2.3–4.8)**	5.23 (4.01–6.46)	**6.89 (4.7–8.01)**	< 0.0001	7.12 (5.98–8.4)	7.03 (5.71–8.40)	7.23 (5.5–8.9)
Total Testosterone (ng/ml)	**6.1 (5.4–6.8)**	4.8 (4.4–5.36)	**3.4 (3.1–3.8)**	< 0.0001	3.9 (3.7–4.1)	3.3 (3.0–3.7)	3.6 (3.1–4.2)
TSH (mIU/L)	1.53 (1–2.1)	1.6 (1–2.02)	1.6 (1–2.02)	< 0.0001	3.15 (2.5–3.8)	3.15 (2.5–3.8)	3.15 (2.5–3.8)
Premature Ejaculation Assessments							
PEDT	18.8 (17.8–19.8)	18.8 (11.4–26.1)	18.5 (11.2–5.8)	< 0.0001	7.02 (5.1–9.0)	7.23 (5.4–9.0)	7.4 (6.2–8.6)
Self-estimated IELT (s)	**100 (25–175) #**	114 (40–188) #	**119 (59–179)**	< 0.0001	315 (190–440)	317 (125–510)	314 (188–440)
Depression Assessments							
BDI-II	**45 (35–55) #**	48 (38–58) #	**51 (42–60)**	< 0.0001	8.75 (4.5–13)	8.7 (4.4–13)	8.75 (4.5–13)
Sexual Assessments							
EF	19 (18–20)	19.5 (17–22)	29.5 (29–30)	NS	26.5 (26–27)	27 (26–28)	26.5 (26–27)
OR	5 (3–6)	5 (1–9)	5 (1–9)	NS	5 (3–7)	5 (3–7)	6 (3–9)
SD	4 (2–6)	6 (4–8)	6 (4–8)	NS	5.5 (3–8)	5 (1–9)	6 (4–8)
OS	4(2–6)	4.5 (3–6)	6.5 (5–8)	NS	5 (4–6)	6 (4–8)	4.5 (3–6)
IS	2 (1–3)	3 (1–5)	2 (1–3)	< 0.0001	6 (4–8)	7 (6–8)	6 (3–9)
IIEF-15- Score	**34 (26–41)**	**38 (26–58)**	**49 (33–58)**	< 0.0001	48 (40–56)	50 (40–60)	49 (39–59)

*PE* Premature Ejaculatory dysfunction; *DM* Diabetes mellitus type II *BMI* Body mass index; *TSH* Thyroid stimulating hormone; *PEDT* Premature ejaculation diagnostic tool; *IELT* Intravaginal ejaculatory latency time; *BDI-II* Beck’s Depression Inventory II; *EF* Erectile function; *OR* Orgasmic function; *SD* Sexual desire; *OS* Overall satisfaction; *IS* Intercourse satisfaction; *IIEF-15* International index of Erectile Dysfunction-15. Kruskal-Wallis test was used to assess the distribution of values across the subgroups based on the length of CAG repeats

Data are presented as median (interquartile range). Significant differences among PE + DM subgroups are shown in bold (*P* < 0.001). # (*P* < 0.05) shows that a result is significantly different from only one of the other PE + DM subgroups. *p < 0.0167 (Kruskal-Wallis/two tailed test with Bonferroni correction) means differences between PE + DM subgroups and entire control group. No statistical difference was found between 3 subgroups (the short, medium, and long CAG) of controls

**Fig. 2:**
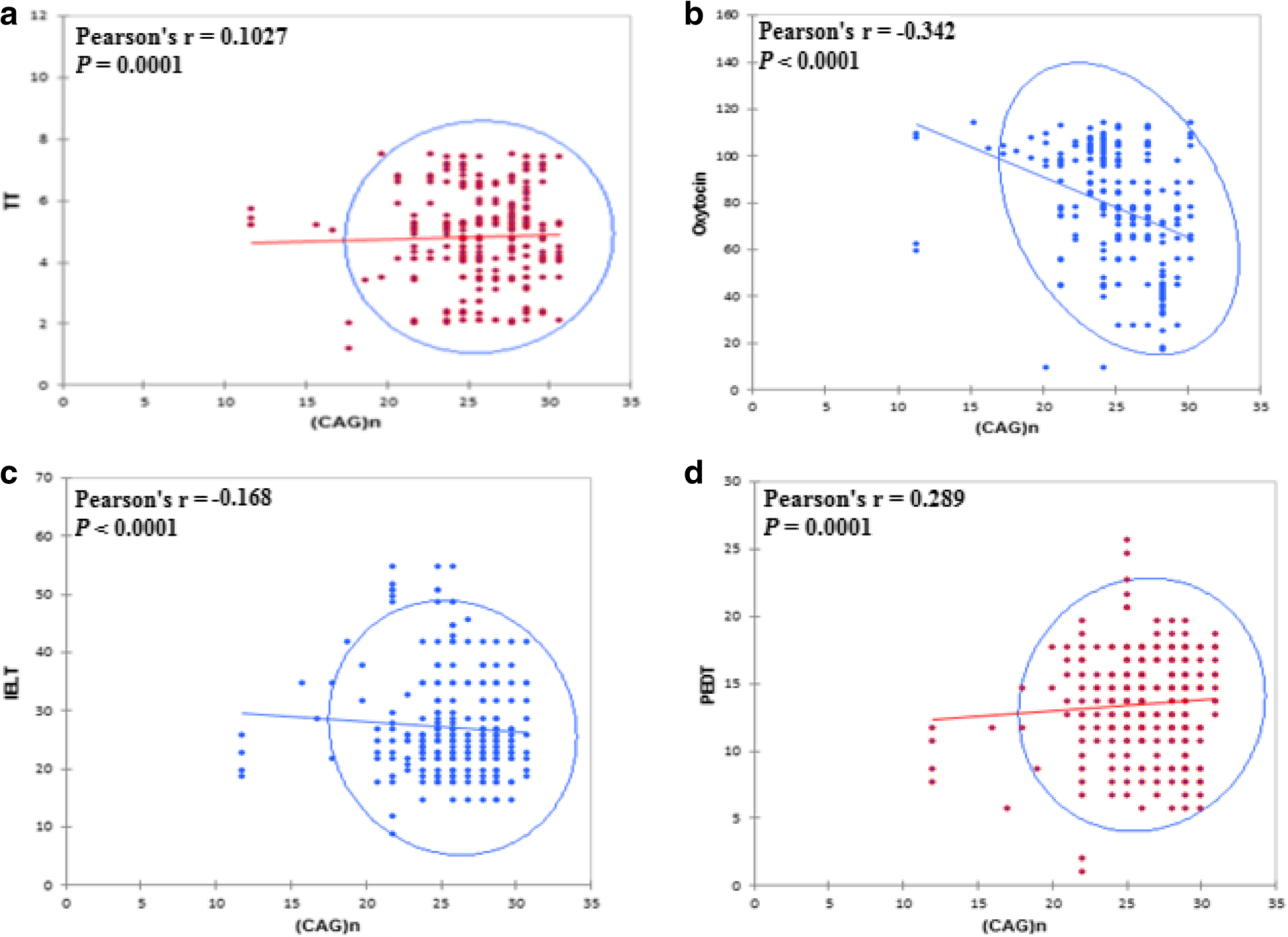
Statistically significant effect of length variations of Androgen receptor (*CAG*)n repeats polymorphism on hormonal profile and sexual parameters among diabetic Premature Ejaculatory dysfunction patients. Scatter graph shows linear relationship between (**a**) (*CAG*)n repeats numbers and testosterone (TT) (**b**) (*CAG*)n repeats numbers and oxytocin (**c**) (*CAG*)n repeats numbers and Intravaginal ejaculatory latency time (IELT) (**d**) (*CAG*)n repeats numbers and Premature ejaculation diagnostic tool (PEDT). While solid line represents regression line

The significant effect of length variations of the androgen receptor (*CAG*)n repeats polymorphism on hormonal profile and sexual parameters was depicted in Fig. 2 which shows that the long (*CAG*)n repeats have a positive correlation with testosterone and negative correlation with oxytocin. Among the analyzed PE patients, no statistically significant difference was found for the association of different (*GGC*)n polymorphic repeats length with oxytocin, total testosterone, TSH and IIEF-15- Score (Table 3).

**Table 3:** Clinical and sexual parameters of subjects with PE + DM and normal group based on Androgen receptor (*GGC*)n repeats

Subjects with PE + DM *n* = 250				Control subjects *n* = 125		
	GGC Long stretches (≥26) *n* = 42	GGC Medium stretches (22–25) *n* = 110	GGC short stretches (≤21) *n* = 98	GGC Long stretches (≥26) *n* = 31	GGC Medium stretches (22–25) *n* = 79	GGC short stretches (≤21) *n* = 40
Clinical Parameter						
Age (years)	45 (35–55)	43.5 (32–58)	45 (30–60)	45 (30–60)	46 (32–60)	45 (30–60)
BMI (Kg/m2)	32.45 (30.8–34.1)	32.25 (30.5–34.0)	31 (30–32)	31.25 (28.9–33.6)	30.8 (27.9–33.7)	31.9 (28.85–35.01)
Blood Sampling Time (8–10 Am)	9.50 (9.00–10	9.57 (9.15–10)	8.72 (8.15–9.30)	9.00 (8.00–10)	8.65 (8.00–9.30)	8.80 (8.30–9.30)
Diabetes duration (years)	9.3 (4.21–14.56)	7.85(2.2–13.5)	8.30 (4.5–12.1)	9.67 (6.37–12.98)	8.4 (4.4–12.5)	7.5 (3.3–11.8)
HbA_1C_ (%)	7.05 (6.2–7.9)*	7.2 (6.4–8.00)*	7.1 (6.3–7.9)*	4.2 (3.9–4.6)	4.1 (3.7–4.5)	4.1 (3.4–4.9)
Hormonal Assays						
Oxytocin (pg/ml)	79.1 (51.9–106.3)	82.85 (65.9–99.8)	83.8 (68.4–97.8)	79.35 (76.5–82.2)	80 (78.9–81.1)	79.5 (77.9–81.2)
Prolactin (ng/ml)	5.00 (4.01–5.98)	5.11 (4.34–5.95)	5.01 (4.1–6.00)	7.2 (5.6–8.99)	7.1 (5.8–8.50)	6.95 (4.9–8.96)
Total Testosterone (ng/ml)	4.9 (3.8–5.9)	4.8 (4.4–5.36)	4.8 (3.8–5.8)	5.1 (3.4–6.9)	4.1 (3.9–4.4)	4.9 (4.1–5.8)
TSH (mIU/L)	2.7 (2.5–2.9)	2.5 (2.1–2.9)0	3.0 (2.5–3.5)	2.9 (2.1–3.8)	3.1 (2.5–3.8)	2.9 (2.1–3.8)
Premature Ejaculation Assessments						
PEDT	22.5 (20–25.1)*	21.9 (17.8–26)*	21.3 (16.8–25.9)*	7.7 (6.57–9.00)	7.4 (6.8–8.19)	7.6 (6.2–9.00)
Self-estimated IELT (s)	168 (156–180)*	170 (165–175)*	168 (158–178)*	344 (201–488)	342 (224–460)	343 (250–436)
Depression Assessments						
BDI-II	49 (40–58)*	48 (38–58)*	51 (42–60)*	10 (7–13)	11 (9–13)	11 (9–13)
Sexual Assessments						
EF	29.5 (29–30)	29.5 (29–30)	27 (26–28)	27 (26–28)	26.5 (26–27)	26.5 (26–27)
OR	3 (1–5)	3 (1–5)	5 (3–7)	5 (3–7)	6 (3–9)	6 (3–9)
SD	7 (6–8)	8 (7–9)	5 (1–9)	5 (1–9)	6 (4–8)	6 (4–8)
OS	4.5 (3–6)	4.5 (3–6)	6 (4–8)	6 (4–8)	4.5 (3–6)	4.5 (3–6)
IS	3 (1–5)*	3 (1–5)*	6 (4–8)	7 (6–8)	6 (3–9)	6 (3–9)
IIEF-15- Score	47 (40–54)	48 (41–55)	49 (39–60)	50 (40–60)	49 (39–59)	49 (39–59)

*PE* Premature Ejaculatory dysfunction; *DM* Diabetes mellitus type II; *BMI* Body mass index; *TSH* Thyroid stimulating hormone; *PEDT* Premature ejaculation diagnostic tool; *IELT* Intravaginal ejaculatory latency time; *BDI-II* Beck’s Depression Inventory-II; *EF* Erectile function; *OR* Orgasmic function; *SD* Sexual desire; *OS* Overall satisfaction; *IS* Intercourse satisfaction; *IIEF-15* International index of Erectile Dysfunction-15

Data is presented as median (Interquartile range). Values in (*) *p* < 0.05 are comparison with the entire control

### Association of trinucleotide repeats with sexual parameters

Studying the correlations between (*CAG*)n repeats and the other variables in the patients’ group shows that the highest numbers of (*CAG*)n repeats are related to the highest testosterone levels, the lowest oxytocin levels, the highest PEDT and the lowest IELT, BDI-II, IS, and IIEF-15 scores. When the same study is performed with the number of (*GGC*)n repeats, no significant correlations are observed with the hormonal variables, but significant correlations are still found with PEDT and IELT (i.e. indicators of premature ejaculation), and BDI-II (indicator of depression), not with IIEF-15 score (indicator of erectile function) (see Table 4). Oxytocin and testosterone were inversely correlated with self-estimated IELT (*r* = − 0.019, *p* = 0.0001; *r* = − 0.024, *p* = 0.0001) and IIEF-15 (*r* = − 0.302; *p* = 0.0001, *r* = − 0.009 *p* = 0.001). Based on this finding oxytocin and testosterone are potent predictors of sexual function (Table 5).

**Table 4:** Correlation between tri-nucleotide repeats, clinical variables, and sexual parameters in whole samples

Variables	Hormonal Parameters				Sexual parameters								
	Oxytocin	Prolactin	TSH	TT	Self- estimated IELT	PEDT	BDI	EF	OR	SD	IS	OS	IIEF-15
(CAG)n	*r* = **− 0.342** ρ = < 0.0001	*r* = − 0.065 ρ = 0.303	*r* = − 00.536 ρ = 0.401	*r* = **0.1027** ρ = < 0.0001	*r* = **− 0.168** ρ = < 0.0001	r = **0.289** ρ = < 0.0001	*r* = − **0.259** ρ = < 0.0001	*r* = − 0.431 ρ = 0.89	*r* = − 0.441 ρ = < 0.92	r = − 0.253 ρ = 0.35	*r* = − **0.029** ρ = < 0.0001	*r* = 0.121 ρ = 0.59	*r* = **− 0.368** ρ = 0.001
(GGC)n	*r* = − 0.312 ρ = 0.231	*r* = − 0.055 ρ = 0.388	*r* = − 0.075 ρ = 0.235	*r* = 0.020 ρ = 0.758	*r* = **− 0.190** ρ = < 0.0001	r = **0.240** ρ = < 0.0001	*r* = **− 0.231** ρ = < 0.0001	*r* = 0.476 ρ = < 0.11	*r* = 0.491 ρ = 0.020	r = 0.312 ρ = 0.021	*r* = 0.273 ρ = 0.06	*r* = 0.112 ρ = 0.13	*r* = − 0.281 ρ = 0.45

Pearson correlation analysis was used to analyze the relationship among clinical, hormonal, and genetic variables. Values in bold are different from 0 with a significance level alpha = 0.05

*TSH* Thyroid stimulating hormone; *TT* Total Testosterone, *PE* Premature Ejaculatory dysfunction; *DM* Diabetes mellitus type II; *PEDT* Premature ejaculation diagnostic tool; *BDI-II* Beck’s Depression Inventory-II; *IELT* Intravaginal ejaculatory latency time; *IIEF-15* International index of Erectile Dysfunction-15

**Table 5:** Correlation of mean plasma levels of biochemical indices of PE + DM subjects and ejaculation related parameters

Parameters	Oxytocin	Prolactin	TSH	TT
Self- estimated IELT	*r* = **−0.019** ρ = 0.0001	*r* = 0.121 ρ = 0.055	*r* = − 0.041 ρ = 0.52	*r* = − **0.024** ρ = 0.0001
PEDT	*r* = 0.022 ρ = 0.72	*r* = **− 0.051** ρ = 0.0001	*r* = 0.069 ρ = 0.27	*r* = 0.100 ρ = 0.11
BDI	*r* = 0.066 ρ = 0.29	*r* = 0.015 ρ = 0.81	*r* = − 0.034 ρ = 0.59	*r* = 0.032 ρ = 0.61
EF	*r* = **− 0.396** ρ = 0**.0001**	*r* = − 0.002 ρ = 0.98	*r* = − 0.053 ρ = 0.400	*r* = 0.033 ρ = 0.604
OR	*r* = **0.187** ρ = 0**.003**	*r* = 0.102 ρ = 0.107	*r* = − 0.021 ρ = 0.73	*r* = − 0.109 0.086
SD	*r* = **0.127** ρ = 0**.0001**	*r* = 0.118 ρ = 0.061	*r* = − 0.016 ρ = 0.800	*r* = 0.025 ρ = 0.697
IS	*r* = − 0.043 ρ = 0.49	*r* = 0.064 ρ = 0.31	*r* = − 0.084 ρ = 0.18	*r* = 0.031 ρ = 0.62
OS	*r* = −0.109 ρ = 0.085	*r* = 0.029 ρ = 0.74	*r* = − 0.039 ρ = 0.64	*r* = 0.002 0.97
IIEF-15	*r* = **− 0.302** ρ = 0**.0001**	*r* = 0.091 ρ = 0.152	*r* = − 0.018 ρ = 0.20	*r* = **− 0.009** ρ = 0.001

Pearson correlation analysis was used to analyze the relationship among clinical, hormonal, and genetic variables. Values in bold are different from 0 with a significance level alpha = 0.05. *PE* Premature Ejaculatory dysfunction; *DM* Diabetes mellitus type II; *PEDT* Premature ejaculation diagnostic tool; *BDI-II* Beck’s Depression Inventory-II; *IELT* Intravaginal ejaculatory latency time; *IIEF-15* International index of Erectile Dysfunction-15

**Fig. 3:**
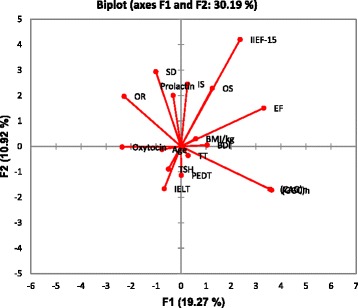
Principal component analysis (PCA) based on clinical, hormonal, and genetic parameters among PE + DM patients. Whereas, correlation circle showing a projection of the initial variables in the factor space. The Coordinate F1 accounts for 19.27% variations and Coordinate F2 accounts for 10.92% variation. PE: Premature Ejaculatory dysfunction; BMI: Body mass index; TSH: Thyroid stimulating hormone; PEDT: = Premature ejaculation diagnostic tool; BDI-II: Beck’s Depression Inventory-II; IELT: Intravaginal ejaculatory latency time; IIEF-15 = International Index of Erectile Dysfunction-15

Another substantial aspect of this study was inspecting the data in such a way as to highlight their similarities and dissimilarities through Principal Component Analysis (PCA) that identify new variables. The plot retained most of the inherent dissimilarities by reducing the dimensionality of the data set and identifying directions known as principal components, which displayed a projection of initial variables in the factor space, along with the variation in the data set were at highest level. By utilizing limited components, comparatively few numbers presented every sample in lieu of values for many variables. The linear combination of the projection of initial variables in the factor space F1 accounts for 19.27% variation and F2 displayed 10.92% variation (Fig. 3).

**Fig. 4:**
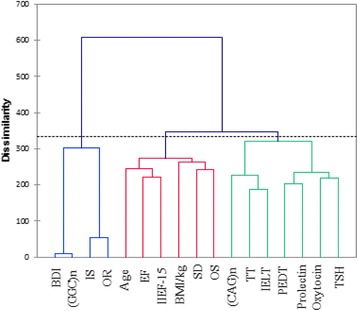
Hierarchical clustering dendrogram shows similarity and dissimilarity with the ordering of cluster among the demographics, Clinical and hormonal profile in PE + DM patients. The dotted line represents the automatic truncation, leading to three clusters; Cluster I (Green): (*CAG*)n repeats, Total testosterone (TT), Intravaginal ejaculatory latency time (IELT), Premature ejaculation diagnostic tool (PEDT), prolactin, Oxytocin and Thyroid stimulating hormone (TSH) Cluster II (Red): Age, Erectile function (EF), International index of Erectile Dysfunction-15 (IIEF-15), Body mass index (BMI), sexual desire (SD) and overall satisfaction (OS) Cluster III (Blue): Beck’s Depression Inventory-II (BDI-II) and(*GGC*)n repeats

While, Cluster Analysis (CA) further evenly aligned all studied parameters into three clusters (Fig. 4). The dendrogram showed the similarity and dissimilarity with the ordering of the cluster among the parameters.

1. **Cluster I (Green):** (*CAG*)n, TT, IELT, PEDT, prolactin, Oxytocin, TSH
2. **Cluster II (Red):** Age, EF, IIEF-15, BMI, SD and OS.
3. **Cluster III (Blue):** BDI-II, (*GGC*)n.

The first cluster is more homogenous and less dissimilar than the second and third cluster which displayed highest variations when looking at class variables. Cluster I have shortest branches displayed a close relationship between BDI-II and (GGC)n repeats. While second and third clusters showed approximately same dissimilarity index.

## Discussion

Sexual function is a complex multidimensional biological process regulating the central dogma of libido and stimulation [26]. It also controls the primary mechanism for the generation of penile tumescence, sexual arousal, rigidity, orgasm, and ejaculation. In the present study, patients do not differ significantly from the controls regarding the number of (*CAG*)n or (*GGC*)n repeats, but those patients with longer trinucleotide repeats have higher testosterone and lower oxytocin levels than other patients and controls. According to the correlation studies, patients with longer trinucleotide repeats have lower IELT and higher PEDT scores than the other patients (i.e. more severe premature ejaculation) and lower IIEF-15 scores i.e. they are more prone to erectile dysfunction.

While, TSH and PRL levels appear to be lower in patients with premature ejaculation and diabetes mellitus than in controls.

Additionally, it is validated that endocrine milieu influences the process of PE and has its effects on the ability to achieve an erection by regulating the pathway of L- arginine-nitric oxide-guanylyl cyclase-cyclic guanosine monophosphate (cGMP) mediated smooth muscle relaxation [27, 28].

This study tends to confirm that long (*CAG*)n repeats in the AR compromise several androgen -dependent functions, especially erectile function. Testosterone is one of the key players in the sexual function by influencing the sexual desire of men through central and peripheral nervous system. The lower levels of serum testosterone (TT) are directly linked to obesity, metabolic syndrome and type 2 diabetes, whereas high levels are in turn associated with PE [29].

In our study, the patients with long (*CAG*)n repeats were found to have lower IIEF- 15 scores than the controls though they had higher testosterone levels. Moreover, an inverse correlation was observed in the patients’ group between (*CAG*)n repeats length and IIEF-15 score. Thus, in patients with high testosterone levels, long (*CAG*)n repeats impair the effect of testosterone on erectile function. This confirms the results of the study by Liu CC et al. (who employed the IIEF 5 instead of IIEF-15) who reported that long (*CAG*)n repeats are an independent risk factor for erectile dysfunction in subjects with testosterone levels above 3.3 ng/mL, but not in those with testosterone levels of 3.3 ng/mL or below [16]. In the PE patients the length of (*CAG*)n repeats was inversely related to self-estimated IELT and positively related to PEDT, i.e. a greater length was associated with more severe PE. The length of (*CAG*)n repeats was also positively related to testosterone levels and negatively related to oxytocin levels.

Regarding the PE patients with long (*CAG*)n repeats, they were found to have higher testosterone levels than the other PE patients and the controls. Testosterone is known to be able to suppress serotonin, a neuromodulator that prevents ejaculations, so that increased testosterone levels could lead to secondary PE. Serotonin is secreted by the brain stem, hypothalamus, and spinal cord and exerts an inhibitory influence on ejaculation. In the patients with long (*CAG*)n repeats, the high testosterone levels that are found could overtake the lowering effect of (*CAG*)n repeats length regarding testosterone action on serotonin, and partly explain why the patients with long CAG repeats suffer from PE as well as those patients with normal or short (*CAG*)n repeats. [30]. Any drug like SSRI, (Selective serotonin reuptake inhibitors) blocks the serotonin reuptake by central neurons successively increased the accumulation of serotonin, which in turn prevents ejaculation and sustained IELT for a bit longer spell [31].

Regarding the PE patients with short (*CAG*)n repeats, they were found to have higher oxytocin levels than the other subgroups of patients and controls. Such higher oxytocin levels could account for the occurrence of PE in these patients with short (*CAG*)n repeats. Oxytocin is a neuromodulator and is involved in the modulation of the symptoms of severe depression. Oxytocin levels may increase during sexual intercourse and facilitate the ejaculation by reducing the ejaculatory latency time [32]. The study performed with mice provided the first empirical evidence that high levels of oxytocin can induce a potent effect on ejaculation by decreasing the IELT and post-copulatory refractory period. Alternatively, rodents with low serum oxytocin levels had prolonged mount and longer refractory spell [33]. Contrarily, in another study, conducted on a small group of active healthy men, the administration of exogenous oxytocin through nasal spray had no substantial outcome on IELT and orgasm [34].

Thus, this study highlights the fact that androgen receptor polymorphism plays a role in PE and that this role is strongly dependent on hormonal cofactors, especially testosterone and oxytocin: patients with long (*CAG*)n repeats have higher TT levels that can lead to PE, whereas patients with short (*CAG*)n repeats may have a higher TT effect (because of short *CAG* repeats) and higher oxytocin levels, thereby also contributing to occurrence of PE.

The finding of high oxytocin levels in PE + DM patients with short (*CAG*)n repeats lengths has not yet been reported and remains to be further confirmed in a large cohort study. There is no clear explanation for such a result. However, it is clearly known that testosterone and oxytocin have opposite effects in neuropsychiatric disorders, cognitive and behavioral functions that might be based upon a direct inhibition of AR on oxytocin transcription [35]. A recent study has proposed a clear opposing effect of testosterone and oxytocin in the modulation of psychiatric disorders such as bipolar disorder and major depressive disorder in diabetic patients [36, 37]. Short (*CAG*)n repeats would have been expected to increase the effects of testosterone and to decrease oxytocin. The inhibiting effect of testosterone on oxytocin seems to be impaired in these patients with short (*CAG*)n repeats in comparison with controls. It has also been reported that testosterone could exert an indirect role through estradiol, which is a metabolite of testosterone that could stimulate oxytocin release [35]. However, the definite explanation can be given for this finding of high oxytocin levels in patients with short (*CAG*)n repeats by conducting more robust future studies.

The role of (*GGC*)n repeats length appears to be a minor one in comparison with that of (*CAG*)n repeats length. However, there is an inverse significant relationship between the length of (*GGC*)n repeats and self-estimated IELT and a positive significant relationship with PEDT, which means that longer (*GGC*)n repeats have an impact on severe PE.

Furthermore, trinucleotide repeats (TNR) polymorphism findings on depression analysis (BDI-II score), demonstrating that all subgroups (PE + DM) had significantly higher state depression than controls, are in line with previous research stated that both PE and ED were associated with severe free-floating anxiety in DM patients [38, 39]. Other published studies suggested that organic and neurobiological factors implicated more than psychological factors in the development of acquired PE [9, 40]. Rastrelli G et al. stated that hypoprolactinemia has been associated with erectile dysfunction and premature ejaculation, findings further confirmed in the general European population and infertile men. They thought that low PRL might be a mirror of an increased dopaminergic or a decreased serotoninergic tone [41]. Corona G et al. showed that PRL (as well as TSH) levels progressively increased from patients with severe PE towards those with an-ejaculation (and that the opposite was observed for testosterone levels). Interestingly, Corona G. et al. included in their discussion the following statements: ‘The speculation that low basal PRL could mirror an impaired serotoninergic pathway is based also on the observation that… hypoprolactinemia was associated with psychobiological features, often considered associated with a low serotoninergic signaling. These include anxiety symptoms and a major propensity towards metabolic syndrome’ [42]. This appears to be in accordance with the finding in the present study of low PRL levels in patients with PE and type 2 diabetes. This low serotoninergic tone is likely to play a role in PE in such patients. Moreover, Corona et al. [42] give also interesting information regarding TSH and thyroid hormones in PE. Interestingly, the present study also found low TSH levels in PE patients even though we had excluded patients with abnormal TSH levels, unlike Corona’s study (in Corona’s study, 33 /2652 patients suffered from hyperthyroidism).

This lowering effect on TSH levels might be related to the frequent use of metformin that is widely used the drug as the first line of oral hypoglycemic agent for treatment of type 2 diabetes mellitus [43]. Other studies also proposed that metformin has a notable lowering effect on TSH through modification of thyroid hormone receptor binding affinity and by inhibition of AMPK (adenosine 5′ - monophosphate -activated kinase) signaling cascade [44].

The low levels of TSH lead to boost the frequency of contraction in seminal vesicle as well as bulbospongiosus muscles located in the middle of the perineum and are likely responsible for ejaculation with minimal sexual stimulation [45]. Whereas, recovery from low levels of thyreotropin prolonged the emission and expulsion episodes in PE subjects [46, 47]. Consistent with the same scenario another prospective study showed that 50% hyperthyroid cohort had PE and this condition became reversed after therapy, with twofold increased intravaginal ejaculatory latency time [46]. Contrarily, a study on a small group of Dutch patients did not reflect any association between TSH and PE [48].

Typically, a multivariate analysis is the crucial stepping stone towards depicting a scenario between the parameters which are often evident in the classic to the parametric system. For instance, Cluster analysis (CA) that separates all the 16 parameters into three clusters and reveals a heterogeneity among observed parameters, which are assorted into varied clusters. Furthermore, CA also shows a close link among nearby, associated variables. In this framework, the first cluster assorted (*CAG*)n, TT, IELT, PEDT, prolactin, Oxytocin, and TSH from the rest of parameters. This is in agreement with contemporary research evidence, has been described by Humble MB et al., that neurohypophyseal hormones are directly involved in the orgasm function, penile erection, and social bonding. They also facilitate the ejaculation which makes them potential candidates as targets for drug therapy in PE subjects [49].

The novel finding of this work is reporting an association between longer (*CAG*)n homopolymeric tracts and IIEF-15 score. This observation was clearer in CA and PCA analysis which also presented a close liaison between (*CAG*)n, (*GGC*)n and IIEF-15 score. High linkage distance between the cluster I and III indicates that hormonal status of oxytocin and prolactin is not dependent on the length of polymorphic (*GGC*)n repeats of the androgen receptor. This lack of correlation between (*GGC*)n repeats size and hormonal status in PE subjects have also been described previously by Kalliolia E et al. [50].

PCA analysis projects the biochemical and genetic variables in the first two PCAs space exhibited that concentration of prolactin was positively correlated with orgasm function. Whereas, the second group exposed a close affiliation between self-reported intravaginal ejaculatory latencies, the total serum concentration of testosterone, thyrotropin levels, overall sexual satisfaction, premature ejaculation diagnostic tool (PEDT), and with BMI. Interestingly, PCA analysis has also exhibited the correlation between PE parameters (IELT, PEDT, and OS) and hormonal milieu (TT and TSH). The close relationship between TT and IELT suggests that T might have both exogenous and endogenous role on the ejaculatory mechanism through supraspinal control or spinal nucleus of the bulbocavernosus mediated ejaculatory reflex (SNB) [51].

One of the main limitations of our study is that most of the participants filled the questionnaires in front of investigators, which might have triggered uncomfortable feelings, resulting in a negative impact on the precision of the results. Secondly, 250 subjects with PE + DM and 150 healthy men were registered in this study; the small sample size might have influenced the outcomes. Third, we do not record the stopwatch measured IELT values in this study, which might affect the exactitude of results. Fourth, certification of the Chinese version of IIEF-15 has not been done before, though it was employed in this study. Fifth, Type 2 DM patients using metformin might have a lowering effect on the thyreotropin levels.

## Conclusion

This study indicates that androgen receptor polymorphism modulates the endocrine effect on ejaculatory reflex and depends strongly on its ‘cofactors’. Moreover, our results also confirmed an association between long tri-nucleotide repeats of androgen receptor, sex steroids, pituitary, and thyroid hormones in relation to acquired premature ejaculatory dysfunction in diabetic type II patients. However, endocrine regulation of PE reflex is a complex phenomenon that requires further investigation.

## Abbreviations

AR: Androgen receptor
BDI-II: Beck Depression Inventory-II
DBD: DNA-binding domain
DM: Diabetes mellitus
EF: Erectile function
FSH: Follicular stimulating hormone
IELT: Self-reported Intra-Vaginal Ejaculatory Latency Time
IIEF-15 score: International Index of Erectile Function-15
IS: Intercourse satisfaction
ISSM: International Society for Sexual Medicine
LBD: Ligand binding domain
NTD: N-terminal transactivation domain
OR: Orgasmic function
PE: Premature ejaculation
PEDT: Premature ejaculation diagnostic tool
SD: Sexual desire
TNR: Tri-nucleotide repeat
TT: Testosterone
